# Prevalence and predictors of Toxoplasma gondii infection in pregnant women from Dhamar, Yemen

**DOI:** 10.1186/s12879-019-4718-4

**Published:** 2019-12-30

**Authors:** Abdulelah H. Al-Adhroey, Amat Al-Khaleq O. Mehrass, Abdulqawi A. Al-Shammakh, Abdullatif D. Ali, Mohammed Y. M. Akabat, Hesham M. Al-Mekhlafi

**Affiliations:** 1 Department of Community Medicine, Faculty of Medicine and Health Sciences, Thamar University, PO Box 87246, Dhamar, Yemen; 2 Department of Gynecology and Obstetrics, Faculty of Medicine and Health Sciences, Thamar University, Dhamar, Yemen; 3 Department of Biochemistry, Faculty of Medicine and Health Sciences, Thamar University, Dhamar, Yemen; 4 Medical Research Center, Jazan University, Jazan, Kingdom of Saudi Arabia; 5 Department of Parasitology, Faculty of Medicine and Health Sciences, Sana’a University, Sana’a, Yemen

**Keywords:** Toxoplasmosis, Predictors, Pregnant women, Prevalence, *Toxoplasma gondii*, Yemen

## Abstract

**Background:**

Toxoplasmosis is a common and serious parasitic infection caused by the ubiquitous obligatory intracellular protozoan organism, *Toxoplasma gondii*. Although infection with *T. gondii* is usually asymptomatic in healthy individuals, it can lead to severe pathological effects in congenital cases and immunocompromised patients. This study aimed to determine the seroprevalence of *T. gondii* and its predictors among pregnant women seeking prenatal and medical care at the general maternal and child health facility in Dhamar district of Dhamar governorate, Yemen.

**Methods:**

A total of 420 pregnant women were randomly selected for this cross-sectional study. Participants were screened for anti-*T. gondii* antibodies (i.e. immunoglobulin M; IgM and immunoglobulin G; IgG) using electrochemiluminescence immunoassay. Demographic, socioeconomic, obstetric and behavioural data were collected using a pretested questionnaire via face-to-face interview. Univariate and multivariate analyses were used to identify the independent predictors of *T. gondii* seroprevalence.

**Results:**

The overall seroprevalence of anti-*T. gondii* antibodies (IgG and/or IgM) among the participants was 21.2% (89/420; 95% CI = 17.3–25.1). Anti-*T. gondii* IgG antibodies were detected in 20.0% (84/420) of the women of which 12.9% (54/420) were positive for only IgG and 7.1% (30/420) were positive for both IgG and IgM antibodies. Moreover, 5 women (1.2%) were reactive only for IgM antibodies. Significant associations between *T. gondii* seroprevalence and history of spontaneous abortion (*P* <  0.001), raw vegetables consumption (*P* = 0.036), and presence of cats in household (*P* = 0.049) were reported. Multivariate analysis confirmed that history of spontaneous abortion (AOR = 4.04; 95% CI = [2.46, 6.63]) and presence of cats in household (AOR = 1.77; 95% CI = [1.02, 3.07]) are significant predictors of *T. gondii* seroprevalence among the studied participants.

**Conclusion:**

The study found a high seroprevalence (21.2%) of *T. gondii* infection during pregnancy in Dhamar district, which is significantly associated with adverse pregnancy outcomes. The provision of adequate maternal healthcare and health education pertaining to the prevention of *T. gondii* infection is therefore imperative to curtail the prevalence of infection among the studied population.

## Background

Toxoplasmosis is a prevalent zoonotic disease caused by the *Toxoplasma gondii* protozoan parasite [1]. It is estimated that one third of the world’s human population is exposed to this obligate intracellular protozoan [2, 3]. Various mammals, including humans, serve as the intermediate host of *T. gondii* infection, while domestic cats and other members of the family *Felidae* serve as the definitive host. Therefore, humans can be infected through the ingestion of food or water contaminated with definitive host’s faeces that contain *T. gondii* oocysts or through the ingestion of cysts in the meat of intermediate hosts such as sheep, pigs, and birds. Women infected with *T. gondii* before pregnancy usually do not transmit the parasite to their foetuses [4]. Acute toxoplasma infection during pregnancy, which is transmitted vertically, can lead to adverse outcomes for the foetus and newborns, including foetal loss or serious congenital anomalies [5]. Moreover, toxoplasmosis is an opportunistic infection that can cause severe complications in immunocompromised persons [6, 7]. The overall risk that acute *T. gondii* infection could result in a congenital infection is estimated to be about 30%, with higher risks during the third trimester [8]. However, some environmental, behavioural, sociodemographic, and obstetric factors have been suggested as important predictors of *T. gondii* infection. Examples of these factors include geographic location, consumption of contaminated water or undercooked meat, presence of cats in the household, exposure to contaminated soil (through farming or gardening barehanded), history of spontaneous abortion (miscarriage), and older maternal age [9, 10].

Diagnosis of toxoplasmosis is generally dependent on serological and clinical analysis. Although *Toxoplasma*-specific antibodies (immunoglobulins) are detected in the serum of pregnant women within one to 2 weeks of exposure to the infection, the serological results for those immunoglobulins are often incapable of differentiating between acute and chronic infections [11, 12]. Detection of IgM antibodies or both IgM and IgG antibodies indicate an acute infection, while negative results may suggest either a very recent infection or the absence of infection [13, 14]. However, positive IgM results may be due to unreliable commercial test kits or because IgM can remain detectable in serum after an acute infection has ended [15–17]. To increase the sensitivity and specificity of the serological evolution, additional confirmatory assays such as seroconversion and IgG avidity testing should be done [14, 17]. Also, positive polymerase chain reaction for *T. gondii* in the amniotic fluid is expected when abnormal ultrasound findings such as hydrocephalus, microcephaly, and calcifications are observed [12, 18].

In Yemen, the published reports on *T. gondii* infection are entirely serologically-based surveys that indicate varied seroprevalence across the country. The overall seroprevalence of *T. gondii* infection has been reported to range from 14 to 65%, with seroprevalence of acute *Toxoplasma*-specific IgM antibodies ranged from 1.5 to 14% [19–25]. Indeed, there is a great scarcity of information on the risk factors of *T. gondii* infection among pregnant women in Yemen, and data on the burden of toxoplasmosis in Dhamar governorate, for example, are not available. Therefore, this study aimed to determine the seroprevalence and predictors of known *T. gondii* infection among pregnant women in Dhamar district, Yemen.

## Methods

### Study design

A cross-sectional study was carried out in Dhamar district between December 2014 and June 2015. Data were collected by trained health personnel from the general maternal and child health care clinics using a pretested questionnaire constructed in English and translated into Arabic, the local language. At the clinics, pregnant women undergo a variety of prenatal examination including physical examination, medical history, laboratory testing and ultrasound scans. Thus, this study did not involve blood collection and rather depend on results by a private laboratory contracted by the clinics (permission was acquired). ‘Known *T. gondii* infection’ was defined as a woman with documented, physician-diagnosed toxoplasmosis and/or laboratory results for the serological examination of *T. gondii* infection. Toxoplasmosis was recognized by the participants as ‘*Al-Gerthoumah*’, a local name, which means ‘the germ causing abortion’.

### Study setting

**Fig. 1:**
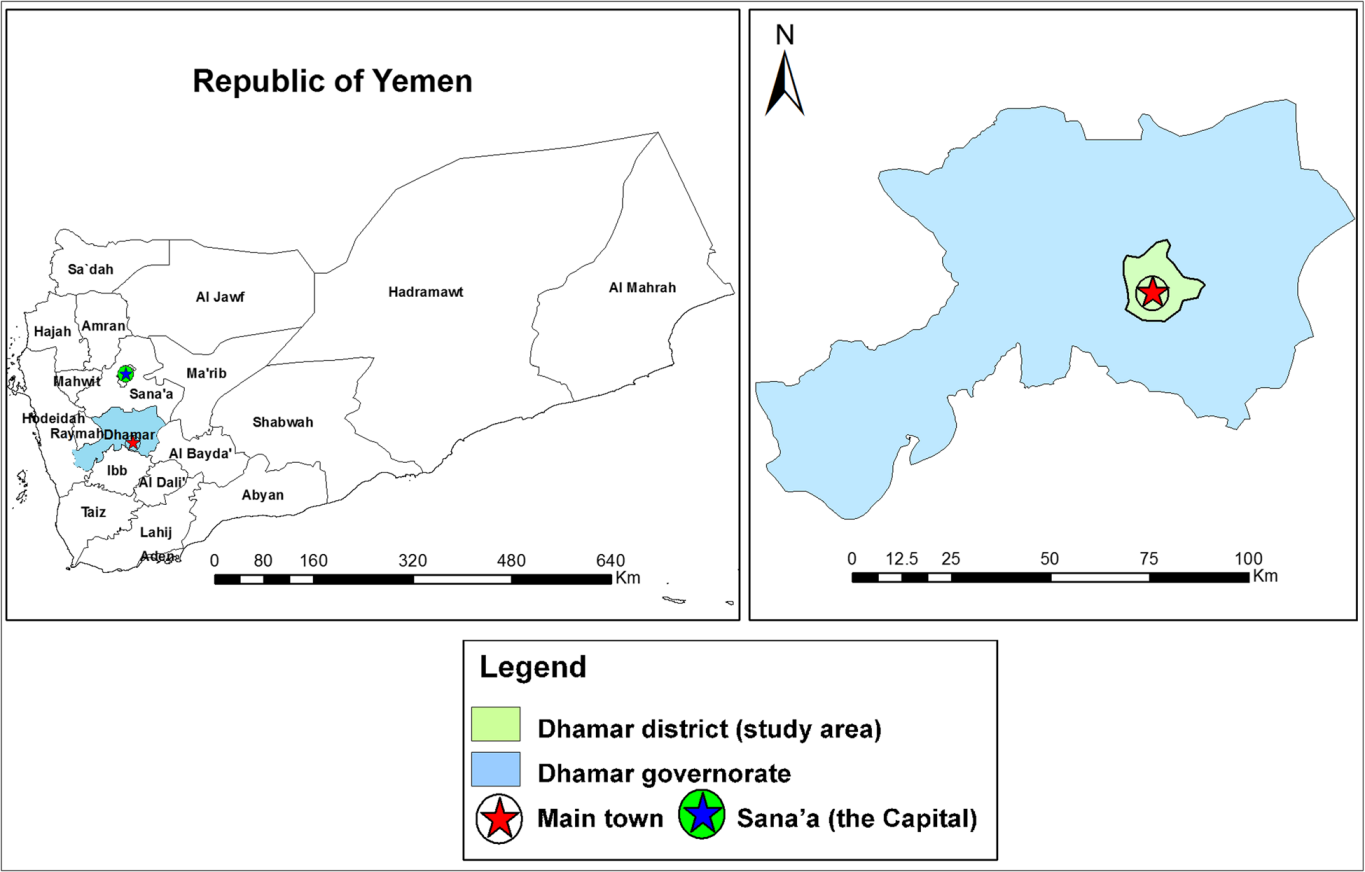
Map showing location of Dhamar governorate and Dhamar district, Yemen (study area). The map was created using the Esri ArcMap 10.7 software

The study was conducted in Dhamar district (44.17 °E, 14.67 °N) in Dhamar governorate, which is located to the south of the capital city of Yemen, Sana’a (Fig. 1). The weather is temperate, the economy is mostly agricultural, and the total population is about 256,000. Dhamar district comprises an urban city, called Dhamar, and 14 rural catchment villages. The study setting was the general maternal and child health facility of the district. The facility has prenatal, reproductive, and outpatient medical care clinics, and it is where the majority of women of child-bearing age in this locality seek health care.

### Sample size and study population

The sample size required for this study was calculated according to the WHO practical manual for the determination of sample size in health studies [26]. Based on a 95% confidence level, a desired precision of 0.05, and an expected toxoplasmosis seroprevalence of 50% because no data are available from Dhamar governorate, the minimum sample size required was estimated as 384 pregnant women. To take account of non-response rate, the sample size was inflated by 10% to get a total sample size of 422.

**Fig. 2:**
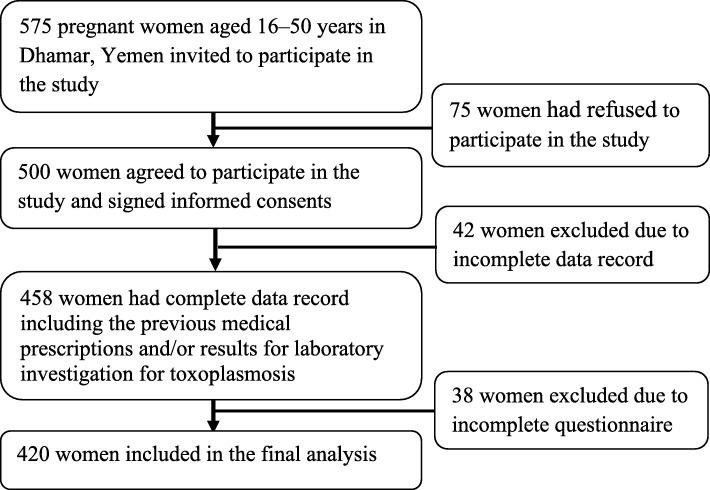
Flowchart of the participation in the study

The participants were randomly selected from the apparently healthy pregnant women attending the targeted prenatal and medical clinics. Out of 575 pregnant women invited to take part in this study, 500 women aged 16–50 years had agreed voluntarily to participate in the survey. Of these 500 women, 420 women completed the questionnaire and were included in the final analysis (Fig. 2).

The study protocol was approved by the Thamar University Medical Ethics Committee, Dhamar, Yemen. A clear description about the aim of the study and the nature of participation was provided to the participants before commencing data collection. The participants were informed that their identity and personal data would be kept strictly confidential and their participation was voluntary. Written consent could not be obtained from this community due to gender-sensitive issues. Therefore, informed verbal consent was taken from each participant and this procedure was approved by the Medical Ethics Committee.

### Data collection

The pregnant women were interviewed to fill in a pre-tested questionnaire for their sociodemographic, obstetric and behavioural characteristics. Information on the following sociodemographic variables was obtained from the participants: maternal age, education level (no formal education (i.e. < 6 years of formal education) versus primary school or above equivalent to ≥6 years of formal education), place of residence (urban area versus rural area of Dhamar district (i.e. those who are living in the rural catchment villages of the district)), and household monthly income. Moreover, information on the following obstetric variables was also obtained: age at marriage, number of children, history of spontaneous abortion, history of giving birth to a deformed baby, and history of blood transfusion. In addition, information on behavioural characteristics including working on a farm, type of drinking water source for the household, consuming raw vegetables, consuming raw milk, chewing khat (the green leaves of the shrub *Catha edulis*, which are commonly chewed in Yemen and East Africa for social and psychological reasons), handling fresh meat such as mutton, lamb, beef, or minced-meat products, and presence of cats or other domestic animals (such as dogs, cattle, sheep, goats, donkeys and chickens) at the household were also collected. Type of drinking water sources was classified into improved sources (i.e. piped water supply) and unimproved sources (i.e. wells, streams, dams, and rain), according to the criteria of WHO/UNICEF [27].

### Serological test for ***T. gondii*** antibodies

About 5 mL venous blood samples were collected from pregnant women seeking healthcare at the general maternal and child health care clinics. The serological test for the presence of anti-*T. gondii* IgG and IgM antibodies was carried out by a private laboratory using an electrochemiluminescence immunoassay (Roche Diagnostics, Mannheim, Germany) according to manufacturer’s instructions. The sera were considered positive if IgG and/or IgM antibodies were detected following the cut-off values provided by the manufacturer. Although indirect Enzyme Linked Immunosorbent Assay (ELISA) was found to be the most frequent serological method used for screening of toxoplasmosis in pregnant women [28], electrochemiluminescence immunoassay was found more sensitive and specific than ELISA [29, 30].

### Data analysis

IBM SPSS statistical software, version 22.0, was used in the statistical data analysis. The study variables were dichotomously presented as a percentage and the 95% confidence intervals (CI) for proportion. For inferential statistics, the seroprevalence of *T. gondii* infection was considered as the dependent variable and demographic, socioeconomic, obstetric and behavioural factors were considered as the explanatory variables. Pearson’s Chi square test was performed to assess the differences between groups, and Fisher’s exact test was used when the number of expected observations in one or more cells in a 2 × 2 contingency table is less than 5. where applicable. To ascertain the independent effect of each explanatory variable, all variables that showed a *P* value of ≤0.25 in the univariate analysis were used to develop a multivariate logistic regression model as suggested by Bendel and Afifi [31]. The odds ratios (OR) and the corresponding 95% CI were calculated by using univariate and multiple logistic regression. A *P* value of < 0.05 was considered as the level of statistical significance for all tests.

## Results

### General characteristics of participants

A total of 500 pregnant women aged between 16 and 45 years agreed to participate in the survey. However, due to missing data, 420 completed questionnaires were considered in the data analysis. Early marriage is a common practice in the study areas, with 42.4% (178/420) of the participants were married before the age of 18. Moreover, 34.5% (145/420) of participants had a history of spontaneous abortion. With regards to the gestational age, it was found that 20.2% of the women were in the first trimester of pregnancy, 35.7% were in the second trimester and 44.1% were in the third trimester. In addition, about one third (36.9%) and two thirds (67.6%) of the participants had no formal education and a low household monthly income (< US$200/month; US$1 = YER216), respectively. The general demographic and socioeconomic characteristics of these participants are presented in Table 1.

**Table 1:** General characteristics of the pregnant women in Dhamar, Yemen (*n* = 420)

Characteristics	n (%)
Age groups (years)	
≥ 25	210 (50)
< 25	210 (50)
Age at marriage (years)	
< 18	178 (42.4)
≥ 18	242 (57.6)
Gestational age	
First trimester	85 (20.2)
Second trimester	150 (35.7)
Third trimester	185 (44.1)
Number of children	
≥ 3	136 (32.4)
< 3	284 (67.6)
Residence	
Rural	182 (43.3)
Urban	238 (56.7)
Socioeconomic status	
No formal education	155 (36.9)
Low household monthly income (< US$ 200)	284 (67.6)
Presence of improved drinking water sources	239 (56.9)
Presence of cats in household	95 (22.6)
Presence of domestic animals at household	192 (45.7)

All values are number (%). *US$* United States Dollar (US$1 = YER216)

### Prevalence and factors associated with *T. gondii* infection

The overall seroprevalence of anti-*T. gondii* antibodies (IgG and/or IgM) among the participants was 21.2% (89/420; 95% CI = 17.3–25.1). It was found that 12.9% (54/420) of the women were reactive only for IgG antibodies, 7.1% (30/420) were reactive for both IgG and IgM antibodies, and 1.2% (5/420) were reactive only for IgM antibodies.

Table 2 shows the associations between the prevalence of infection and some demographic, socioeconomic, behavioural and obstetric factors. It was found that the pregnant women who were ≥ 25 years old showed a potentially higher seroprevalence of *T. gondii* infection compared to those aged below 25 years (24.8% vs. 17.6%; *P* = 0.073). Similarly, women who had more than three living children had higher seroprevalence of *T. gondii* infection than their counterparts (25.0% vs. 19.4%); however, the difference was not statistically significant (*P* = 0.186).

**Table 2:** Univariate analysis of factors associated with *T. gondii* seroprevalence among the pregnant women in Dhamar, Yemen (*n* = 420)

Variables	*Toxoplasma* seroprevalence			
	N participants	Prevalence %	Crude OR (95% CI)	*P*-value^a^
Age (years)				
< 25	210	17.6	1	
≥ 25	210	24.8	1.54 (0.96, 2.47)	0.073
Age at marriage (years)				
< 18	178	22.5	1	
≥ 18	242	20.2	0.88 (0.55, 1.40)	0.582
Number of children				
< 3	284	19.4	1	
≥ 3	136	25.0	1.39 (0.85, 2.26)	0.186
Gestational age				
First trimester	85	18.8	1	
Second trimester	150	21.3	1.21 (0.71, 1.68)	0.647
Third trimester	185	22.2	1.31 (0.61, 2.54)	0.532
History of spontaneous abortion				
No	275	13.1	1	
Yes	145	36.6	3.83 (2.35, 6.22)	< 0.001^**^
History of giving birth to a deformed baby				
No	401	20.9	1	
Yes	19	26.3	1.35 (0.47, 3.85)	0.576
History of blood transfusion				
No	400	21.8	1	
Yes	20	10.0	0.40 (0.10, 1.76)	0.165
Education level				
Primary school or above	265	19.2	1	
No formal education	155	24.5	1.36 (0.85, 2.20)	0.202
Residence				
Urban	238	19.3	1	
Rural	182	23.6	1.29 (0.81, 2.07)	0.285
Household monthly income				
≥ US$ 200	136	20.6	1	
< US$ 200	284	21.5	1.06 (0.64, 1.75)	0.834
Working on a farm				
No	335	21.2	1	
Yes	85	21.2	1.00 (0.56, 1.79)	0.997
Source of drinking water				
Improved (piped water)	239	20.9	1	
Unimproved (wells, streams, dams and rain)	181	21.5	1.04 (0.65, 1.66)	0.876
Raw vegetables consumption				
No	24	4.2	1	
Yes	396	22.2	6.57 (1.01, 42.34)	0.036^**^
Raw milk consumption				
No	281	19.6	1	
Yes	139	24.5	1.33 (0.82, 2.16)	0.249
Handling fresh meat				
No	18	5.6	1	
Yes	402	21.9	4.76 (0.63, 39.30)	0.076
Khat chewing				
No	214	20.1	1	
Yes	206	22.3	1.14 (0.72, 1.83)	0.575
Presence of cats in household				
No	325	19.1	1	
Yes	95	28.4	1.68 (1.01, 2.85)	0.049^**^
Presence of domestic animals at household				
No	228	18.0	1	
Yes	192	25.0	1.52 (0.95, 2.43)	0.080

*US$* United States Dollar (US$1 = YER216)

Reference group marked as OR = 1; *OR* Odds ratio; *CI* Confidence interval

^a^ Level of significance was determined by Pearson’s Chi Square test or Fisher’s exact test. ^**^ Significant association (*P* < 0.05)

As for the obstetric variables, the pregnant women who had a spontaneous abortion history showed a significantly higher seroprevalence of *T. gondii* infection (36.6% vs. 13.1%; *P* <  0.001). Similarly, the seroprevalence of *T. gondii* infection was found to differ significantly with raw vegetables consumption (22.2% vs. 4.2%; *P* = 0.036) and presence of cats in household (28.4% vs. 19.1%; *P* = 0.049). It was also found that women who declared that they handled fresh meat had a higher seroprevalence of *T. gondii* infection when compared to their peers; however, the difference was only marginally significant (21.9% vs. 5.6%; *P* = 0.076).

Examination of the association between seroprevalence of *T. gondii* infection and the potential predictors using the multivariate logistic regression analysis revealed that a history of spontaneous abortion and the presence of cats in the house were the significant independent predictors for *T. gondii* infection (Table 3). Multivariate regression analysis showed that acquiring *T. gondii* infection increases four times by history of spontaneous abortion (AOR = 4.04; 95% CI = [2.46, 6.63]; *P* <  0.001) and 1.77 times by presence of cats in the house (AOR = 1.77; 95% CI = [1.02, 3.07]; *P* = 0.043). Although statistically significant in the univariate analyses, consumption of raw vegetables was shown to be not a significant predictor of *T. gondii* infection in the multivariate analysis (*P* = 0.059).

**Table 3:** Multivariate analysis of the predictors of *T. gondii* seroprevalence among pregnant women in Dhamar, Yemen (*n* = 420)

Variables	Adjusted OR	95% CI	*P*-value
Age (≥ 25 years)	1.19	0.64, 2.19	0.588
No. of children (> 3)	0.98	0.52, 1.84	0.948
History of spontaneous abortion (yes)	4.04	2.46, 6.63	< 0.001^*^
History of blood transfusion (yes)	0.31	0.07, 1.42	0.130
Education level (no formal education)	1.07	0.62, 1.84	0.813
Handling fresh meat (yes)	4.07	0.50, 35.43	0.241
Raw vegetables consumption (yes)	7.12	0.93, 38,83	0.059
Raw milk consumption (yes)	1.51	0.89, 2.55	0.129
Presence of cats in house (yes)	1.77	1.02, 3.07	0.043^*^
Presence of domestic animals at household (yes)	1.32	0.69, 2.52	0.409

*OR* Odds ratio, *CI* confidence interval

^*^ Significant predictor (*P* < 0.05)

## Discussion

This study is the first to ascertain the prevalence and predictors of known *T. gondii* infection among pregnant women in Dhamar district, Yemen. The overall seroprevalence of *T. gondii* infection reported by this study was 21.2%. This rate is lower than that reported by previous studies conducted in Yemen [19–24]. For instance, 45.4% (269/593) of the pregnant women seeking health care in public and private health facilities in Sana’a city in the north central part of Yemen and 64.3% (431/670) of the pregnant women attending 13 private clinics and hospitals in Aden governorate, southern Yemen, were found seropositive for *T. gondii* infection [23, 24]. On the other hand, a lower seropositive (14.4%) of *T. gondii* infection was reported by a recent study in Hodiedah governorate, western Yemen, among 90 women of childbearing age [25].

When compared to other countries, the seroprevalence reported in this study was in harmony with those reported in Palestine (17.6%), Saudi Arabia (24.1%), Myanmar (30.2%) and Burkina Faso (31.1%) [32–35]. However, higher seroprevalence were reported in other countries such as Brazil (71%), Lebanon (82.6%), Ethiopia (85.3%), and Ghana (92.5%) [36–39]. In contrast, lower seroprevalence of *T. gondii* infection were reported in Zambia (5.9%), Mexico (8.2%), Sri Lanka (12.3%) and in many European countries [2, 40–42]. This variation in the seroprevalence of *T. gondii* infection is observed worldwide and it has been attributed to various determinants such as the use of diagnostic methods of different sensitivity, and the limitations of the surveys in controlling the differences between cohorts [43, 44]. Differences are also attributed to the seroconversion of the short-lived IgM antibodies during pregnancy [45]. For instance, a recent systematic review of the global burden of congenital toxoplasmosis estimated that the proportion of seroconversion of *Toxoplasma*-specific IgM, which showed IgM-positive results, was detectable in only one fifth of pregnant women during their current pregnancy [3]. On the other hand, a recent external assessment of the quality of serological diagnosis for toxoplasmosis in over 843 clinical laboratories in China found that the diagnoses were of high quality, with a mean accuracy of 98% for detecting IgG and 95% for IgM antibodies [45].

The present study showed that 8.3% of the pregnant women had detectable IgM antibodies; 7.1% had anti-*T. gondii* IgM antibodies combined with anti-*T. gondii* IgG antibodies, while 1.2% had IgM antibodies alone. These findings are consistent with previous studies conducted among pregnant women in Sana’a city [23, 46]. However, lower seroprevalence of anti-*T. gondii* IgM antibodies was reported by previous studies among pregnant women in Taiz, southern Yemen [20]. The *Toxoplasma* IgM positive and lgG negative results may indicate an early infection, while the presence of both IgG and IgM antibodies suggests an acute infection [47]. However, further confirmation by using seroconversion and/or IgG avidity tests was not performed by the present study. The presence of IgM antibodies does not always indicate acute infection as IgM may persist positive for up to 18 months in chronic *Toxoplasma* infection [48]. Overall, all previous surveys from Yemen provide no information about seroconversion and IgG avidity testing or clinical diagnosis, which implies that there is major uncertainty regarding the reported estimates.

In line with previous studies around the world, the present survey found that a history of spontaneous abortion and the presence of cats in the household were the independent significant predictors of *T. gondii* infection [19, 49–51]. It is well known that toxoplasmosis during pregnancy has a causal relationship with spontaneous abortion, and it has been suggested that it is associated with IgM seropositivity [52]. It is also well documented that domestic cats are considered as the major contributing factor for *T. gondii* contamination [53]. On the other hand, the univariate analysis showed that the consumption of raw vegetables was also a significant predictor. This is in line with similar results reported among pregnant women in Sana’a, Yemen [23] and elsewhere [50, 54]. The univariate analysis also revealed that maternal age and the handling of fresh meat were marginally significant, a finding that is comparable to those reported in Yemen and Mexico [20, 55]. However, unlike the present study, other reports from Cameroon, Tunisia and Brazil indicate that the consumption of raw or undercooked contaminated meat such as pork, mutton, lamb, beef, or minced-meat products is an independent predictor for acquiring *Toxoplasma* infection [36, 55, 56]. Differences in the seroprevalence may be due to local variations in the practices of cooking meat, types of consumed meat, and burden of *Toxoplasma* infection in the animals consumed [1]. The majority (95.7%) of the participants in the present study reported that they customarily handle fresh meat.

In the present study, the seroprevalence of known *T. gondii* infection was not influenced by the education level, place of residence, household monthly income, age at marriage, number of children, history of giving birth to a deformed baby, gestational age, history of blood transfusion, working on a farm, using unimproved sources for drinking water, drinking raw milk, chewing khat and the presence of domestic animals (excluding cats) at the household. This finding is generally consistent with those reported by previous studies [53, 55, 57]. By contrast, a previous study from Burkina Faso identified urban residence and having at least secondary education level as significant risk factors of *T. gondii* infection among pregnant women attending antenatal care at centers in Bobo-Dioulasso city [35]. In Taiz, Yemen, drinking untreated water has been reported as a significant risk factor for higher *T. gondii* seroprevalence [20]. The difference in results could be attributed to the climatic conditions in Yemen; Dhamar district is located in the mountains and has a cooler and drier environment than that of Taiz, which is warmer and more humid and thus provides a more conducive environment for the oocyst of *T. gondii*, enabling it to remain infectious in water for several months [9].

We acknowledge some limitations that should be considered when interpreting the findings of the present study. First, this study was based on a limited population of pregnant women attending the general maternal and child health facility in Dhamar district. However, it should be noted that this facility is where the majority of women of child-bearing age in Dhamar governorate seek prenatal, reproductive, and medical care. Second, due to the study design (i.e. cross-sectional), it was not possible to infer causality between the *T. gondii* infection and the significant predictors. Also, follow up the participants to detect seroconversion was not performed. Third, known or diagnosed *T. gondii* infection, based on laboratory testing performed by a private laboratory, was considered by this study. However, adopting an inexpensive and non-invasive research methodology has been confirmed to be more convenient in a poor, resource-limited and broken country like Yemen [58].

## Conclusions

This is the first study to reveal the prevalence and predictors of known *T. gondii* infection in Dhamar district, Yemen. About one fifth of the studied population were seropositive for *T. gondii* infection, with presence of cats in the household and a history of spontaneous abortion are significant independent predictors of the infection. Due to the method employed, the present study provides health care personnel in Yemen with an estimate of the magnitude of toxoplasmosis in a vulnerable community. Further population-based surveys, including seroconversion and IgG avidity testing, clinical diagnosis and evaluation of knowledge, attitude and practices towards toxoplasmosis, are recommended so that the burden of this public health problem can be determined more accurately.

## Data Availability

The datasets used and/or analysed during the current study are available from the corresponding author on reasonable request.

## Abbreviations

AOR: Adjusted odds ratio
CI: Confidence interval
ELISA: Enzyme linked immunosorbent assay
IgG: Immunoglobulin G
IgM: Immunoglobulin M
WHO: World Health Organization

## References

[1] Tenter AM, Heckeroth AR, Weiss LM (2000). *Toxoplasma gondii*: from animals to humans. Int J Parasitol.

[2] Pappas G, Roussos N, Falagas ME (2009). Toxoplasmosis snapshots: global status of *Toxoplasma gondii* seroprevalence and implications for pregnancy and congenital toxoplasmosis. Int J Parasitol.

[3] Torgerson PR, Mastroiacovo P (2013). The global burden of congenital toxoplasmosis: a systematic review. Bull World Health Organ.

[4] Montoya JG, Remington JS (2008). Management of *Toxoplasma gondii* infection during pregnancy. Clin Infect Dis.

[5] Kravetz JD, Federman DG (2005). Toxoplasmosis in pregnancy. Am J Med.

[6] Munoz M, Liesenfeld O, Heimesaat MM (2011). Immunology of *Toxoplasma gondii*. Immunol Rev.

[7] Tegegne D, Abdurahaman M, Mosissa T, Yohannes M (2016). Anti-toxoplasma antibodies prevalence and associated risk factors among HIV patients. Asian Pac J Trop Med.

[8] Dunn D, Wallon M, Peyron F, Petersen E, Peckham C, Gilbert R (1999). Mother-to-child transmission of toxoplasmosis: risk estimates for clinical counselling. Lancet.

[9] Yan C, Liang LJ, Zheng KY, Zhu XQ (2016). Impact of environmental factors on the emergence, transmission and distribution of *Toxoplasma gondii*. Parasit Vectors.

[10] Elmore SA, Jones JL, Conrad PA, Patton S, Lindsay DS, Dubey JP (2010). *Toxoplasma gondii*: epidemiology, feline clinical aspects, and prevention. Trends Parasitol.

[11] Many A, Koren G (2006). Toxoplasmosis during pregnancy. Can Fam Physician.

[12] Paquet C, Yudin MH (2013). Toxoplasmosis in pregnancy: prevention, screening, and treatment. J Obstet Gynaecol Can.

[13] Di Carlo P, Romano A, Schimmenti MG, Mazzola A, Titone L (2008). Maternofetal *Toxoplasma gondii* infection: critical review of available diagnostic methods. Infez Med.

[14] Liu Q, Wang ZD, Huang SY, Zhu XQ (2015). Diagnosis of toxoplasmosis and typing of *Toxoplasma gondii*. Parasit Vectors.

[15] Wilson M, Remington JS, Clavet C, Varney G, Press C, Ware D (1997). Evaluation of six commercial kits for detection of human immunoglobulin M antibodies to *Toxoplasma gondii*. The FDA toxoplasmosis ad hoc working group. J Clin Microbiol.

[16] Montoya JG (2002). Laboratory diagnosis of *Toxoplasma gondii* infection and toxoplasmosis. J Infect Dis.

[17] Liesenfeld O, Press C, Montoya JG, Gill R, Isaac-Renton JL, Hedman K (1997). False-positive results in immunoglobulin M (IgM) toxoplasma antibody tests and importance of confirmatory testing: the Platelia Toxo IgM test. J Clin Microbiol.

[18] de Oliveira Azevedo CT, PEAA d B, Guida L, Lopes Moreira ME (2016). Performance of polymerase chain reaction analysis of the amniotic fluid of pregnant women for diagnosis of congenital toxoplasmosis: a systematic review and meta-analysis. PLoS One.

[19] Saif N, Al Ameeri G, Alhweesh M, Alkadasi M, Zaid AA (2014). Seroprevalence of toxoplasmosis in pregnant women in Taiz-Yemen. Int J Curr Microbiol App Sci.

[20] Mahdy MA, Alareqi LM, Abdul-Ghani R, Al-Eryani SM, Al-Mikhlafy AA, Al-Mekhlafi AM (2017). Community-based survey of *Toxoplasma gondii* infection among pregnant women in rural areas of Taiz governorate, Yemen: the risk of waterborne transmission. Infect Dis Poverty.

[21] Alkadasi MN, Putaiah ET, Aameri GA, Sallam A, Olyoa K, Alameri A (2016). Prevalence of toxoplasmosis among pregnant women and risk factors in Al-Kaeda province, Ibb, Yemen. J Bio Innov.

[22] Arwa A, Hmamouch A, Amayour A, Marc I, El Kharrim K, Belghyti D (2015). Epidemiology of toxoplasmosis among married women at birth age in Sana’a City (Yemen). Int J Innov Sci Res.

[23] Al-Eryani SMA, Al-Mekhlafi AM, Al-Shibani LA, Mahdy MMK, Azazy AA (2016). *Toxoplasma gondii* infection among pregnant women in Yemen: factors associated with high seroprevalence. J Infect Dev Ctries.

[24] Muqbil NA, Alqubatii MA (2014). Seroprevalence of toxoplasmosis among women in Aden city, Yemen. Arch Biomed Sci.

[25] Al-Kadassy AM, Baraheem OH, Bashanfer SA (2018). Prevalence of *Toxoplasma gondii* infection in women of child-bearing age in Faculty of Medicine and Health Sciences Hodeida City, Yemen. Pharma Innovation J.

[26] Lwanga SK, Lemeshow S (1991). Sample size determination in health studies: a practical manual.

[27] WHO, UNICEF (2015). Progress on sanitation and drinking-water: 2015 update.

[28] Marques BA, Andrade GMQ, Neves SPF, Pereira FH, Talim MCT (2015). Systematic review of serological methods used in prenatal screening of toxoplasmosis in pregnant women. Rev Med Minas Gerais.

[29] Prusa AR, Hayde M, Unterasinger L, Pollak A, Herkner KR, Kasper DC (2010). Evaluation of the Roche Elecsys Toxo IgG and IgM electrochemiluminescence immunoassay for the detection of gestational *Toxoplasma* infection. Diagn Microbiol Infect Dis.

[30] Firouz ZE, Kaboosi H, Nasiri AF, Tabatabaie SS, Golhasani-Keshtan F, Zaboli FA (2014). Comparative serological study of toxoplasmosis in pregnant women by CLIA and ELISA methods in Chalus City, Iran. Iran Red Crescent Med J.

[31] Bendel RB, Afifi AA (1977). Comparison of stopping rules in forward “stepwise” regression. J Am Stat Assoc.

[32] Nijem KI, Al-Amleh S (2009). Seroprevalence and associated risk factors of toxoplasmosis in pregnant women in Hebron district, Palestine. East Mediterr Health J.

[33] Aqeely H, Eman K, El-Gayar DPK, Najmi A, Alvi A, Bani I (2014). Seroepidemiology of *Toxoplasma gondii* amongst pregnant women in Jazan Province, Saudi Arabia. J Trop Med.

[34] Andiappan H, Nissapatorn V, Sawangjaroen N, Nyunt MH, Lau YL, Khaing SL (2014). Comparative study on *Toxoplasma* infection between Malaysian and Myanmar pregnant women. Parasit Vectors.

[35] Bamba S, Cissé M, Sangaré I, Zida A, Ouattara S, Guiguemdé RT (2017). Seroprevalence and risk factors of *Toxoplasma gondii* infection in pregnant women from Bobo Dioulasso, Burkina Faso. BMC Infect Dis.

[36] Rocha ÉM, Lopes CW, Ramos RA, Alves LC (2015). Risk factors for *Toxoplasma gondii* infection among pregnant women from the state of Tocantins, northern Brazil. Rev Soc Bras Med Trop.

[37] Nahouli H, El Arnaout N, Chalhoub E, Anastadiadis E, El Hajj H (2017). Seroprevalence of anti-*Toxoplasma gondii* antibodies among Lebanese pregnant women. Vector Borne Zoonotic Dis.

[38] Abamecha F, Awel H (2016). Seroprevalence and risk factors of *Toxoplasma gondii* infection in pregnant women following antenatal care at Mizan Aman general hospital, bench Maji zone (BMZ), Ethiopia. BMC Infect Dis.

[39] Ayi I, Edu SA, Apea-Kubi KA, Boamah D, Bosompem KM, Edoh D (2009). Sero-epidemiology of toxoplasmosis amongst pregnant women in the greater Accra region of Ghana. Ghana Med J.

[40] Frimpong C, Makasa M, Sitali L, Michelo C (2017). Seroprevalence and determinants of toxoplasmosis in pregnant women attending antenatal clinic at the university teaching hospital, Lusaka, Zambia. BMC Infect Dis.

[41] Alvarado-Esquivel C, Torres-Castorena A, Liesenfeld O, García-López CR, Estrada-Martínez S, Sifuentes-Alvarez A (2009). Seroepidemiology of *Toxoplasma gondii* infection in pregnant women in rural Durango, Mexico. J Parasitol.

[42] Chandrasena N, Herath R, Rupasinghe N, Samarasinghe B, Samaranayake H, Kastuririratne A (2016). Toxoplasmosis awareness, seroprevalence and risk behavior among pregnant women in the Gampaha district, Sri Lanka. Pathog Glob Health.

[43] Cenci-Goga BT, Rossitto PV, Sechi P, McCrindle CM, Cullor JS (2012). Toxoplasma in animals, food, and humans: an old parasite of new concern. Food Borne Pathog Dis.

[44] Agmas B, Tesfaye R, Koye DN (2015). Seroprevalence of *Toxoplasma gondii* infection and associated risk factors among pregnant women in Debre Tabor, Northwest Ethiopia. BMC Res Notes.

[45] Zhang K, Wang L, Lin G, Sun Y, Zhang R, Xie J, Li J (2015). Results of the national external quality assessment for toxoplasmosis serological testing in China. PLoS One.

[46] Al-Nahari AM, Al-Tamimi AS (2010). Seroprevalence of anti *Toxoplasma gondii* IgG and IgM among pregnant women in Sana'a capital and capital trusteeship. Sci J King Faisal Uni.

[47] Sensini A (2006). *Toxoplasma gondii* infection in pregnancy: opportunities and pitfalls of serological diagnosis. Clin Microbiol Infect.

[48] Zhang K, Lin G, Han Y, Li J (2016). Serological diagnosis of toxoplasmosis and standardization. Clin Chim Acta.

[49] Awoke K, Nibret E, Munshea A (2015). Sero-prevalence and associated risk factors of *Toxoplasma gondii* infection among pregnant women attending antenatal care at Felege Hiwot referral hospital, Northwest Ethiopia. Asian Pac J Trop Med.

[50] Gebremedhin EZ, Tadesse G (2015). A meta-analysis of the prevalence of *Toxoplasma gondii* in animals and humans in Ethiopia. Parasit Vectors.

[51] Halonen SK, Weiss LM (2013). Toxoplasmosis. Handb Clin Neurol.

[52] Dubey JP (2001). Oocyst shedding by cats fed isolated bradyzoites and comparison of infectivity of bradyzoites of the VEG strain *Toxoplasma gondii* to cat sand mice. J Parasitol.

[53] Fallah M, Rabiee S, Matini M, Taherkhani H (2008). Seroepidemiology of toxoplasmosis in primigravida women in Hamadan, Islamic Republic of Iran, 2004. East Mediterr Health J.

[54] Alvarado-Esquive C, Pacheco-Vega SJ, Hernández-Tinoco J, Sánchez-Anguiano LF, Berumen-Segovia LO, FJI R-A (2014). Seroprevalence of *Toxoplasma gondii* infection and associated risk factors in Huicholes in Mexico. Parasit Vectors.

[55] Wam EC, Sama LF, Ali IM, Ebile WA, Aghangu LA, Tume CB (2016). Seroprevalence of *Toxoplasma gondii* IgG and IgM antibodies and associated risk factors in women of child-bearing age in Njinikom, NW Cameroon BMC. Res Notes.

[56] Boughattas S, Ayari K, Sa T, Aoun K, Bouratbine A (2014). Survey of the parasite *Toxoplasma gondii* in human consumed ovine meat in Tunis City. PLoS One.

[57] Alvarado-Esquivel C, Pacheco-Vega SJ, Hernández-Tinoco J, Centeno-Tinoco MM, Beristain-García I, Sánchez-Anguiano LF (2014). Miscarriage history and *Toxoplasma gondii* infection: a cross-sectional study in women in Durango City, Mexico. Eur J Microbiol Immunol (Bp).

[58] Gunaid AA (2002). Prevalence of known diabetes and hypertension in the Republic of Yemen. East Mediterr Health J.

